# A novel gnotobiotic experimental system for Atlantic salmon (*Salmo salar* L.) reveals a microbial influence on mucosal barrier function and adipose tissue accumulation during the yolk sac stage

**DOI:** 10.3389/fcimb.2022.1068302

**Published:** 2023-02-01

**Authors:** Sol Gómez de la Torre Canny, Catherine Taylor Nordgård, Amalie Johanne Horn Mathisen, Eirik Degré Lorentsen, Olav Vadstein, Ingrid Bakke

**Affiliations:** Department of Biotechnology and Food Science, Faculty of Natural Sciences, Norwegian University of Science and Technology, Trondheim, Norway

**Keywords:** gnotobiotic, Atlantic salmon (*Salmo salar* L.), skin mucus, microbiome, yolk sac fry, adipose tissue, intestine, alevin

## Abstract

Gnotobiotic models have had a crucial role in studying the effect that commensal microbiota has on the health of their animal hosts. Despite their physiological and ecological diversity, teleost fishes are still underrepresented in gnotobiotic research. Moreover, a better understanding of host-microbe interactions in farmed fish has the potential to contribute to sustainable global food supply. We have developed a novel gnotobiotic experimental system that includes the derivation of fertilized eggs of farmed and wild Atlantic salmon, and gnotobiotic husbandry of fry during the yolk sac stage. We used a microscopy-based approach to estimate the barrier function of the skin mucus layer and used this measurement to select the derivation procedure that minimized adverse effects on the skin mucosa. We also used this method to demonstrate that the mucus barrier was reduced in germ-free fry when compared to fry colonized with two different bacterial communities. This alteration in the mucus barrier was preceded by an increase in the number of cells containing neutral mucosubstances in the anterior segment of the body, but without changes in the number of cells containing acidic substances in any of the other segments studied along the body axis. In addition, we showed how the microbial status of the fry temporarily affected body size and the utilization of internal yolk stores during the yolk sac stage. Finally, we showed that the presence of bacterial communities associated with the fry, as well as their composition, affected the size of adipose tissue. Fry colonized with water from a lake had a larger visceral adipose tissue depot than both conventionally raised and germ-free fry. Together, our results show that this novel gnotobiotic experimental system is a useful tool for the study of host-microbe interactions in this species of aquacultural importance.

## 1 Introduction

A growing body of evidence has revealed that microbial communities in the intestine and other body sites have a profound effect on human health and disease (Turnbaugh et al., 2007; Aagaard et al., 2013; Ding and Schloss, 2014). These commensal microbial communities have been implicated in metabolic health (Fan and Pedersen, 2021), growth (Schwarzer et al., 2016; Robertson et al., 2019), inflammation and immunity (Clemente et al., 2018; Tilg et al., 2020; Vijay and Valdes, 2022), and nutrient utilization and availability (Sonnenburg and Backhed, 2016; Rowland et al., 2018; Celis and Relman, 2020).

The field of gnotobiotics uses tractable experimental systems that include germ-free (axenic) animals that are raised in the absence of their conventionally associated microbiota (within the limits of detection of tests for identifying microbial contamination), as well as animals to which microbial communities of a defined composition are introduced (Reyniers, 1959). Thus, gnotobiotic animals are an experimental tool to identify biological processes in the host that are mediated by their associated microbiota (Bäckhed et al., 2004), to investigate host responses to colonization with specific microbial isolates or communities of distinct composition (Faith et al., 2014; Schieber et al., 2015), and to validate the function of microbes and microbial communities correlated with host phenotypes and dysbiosis (Smith et al., 2013; Schwarzer et al., 2016).

With more than thirty-six thousand species (Fricke et al., 2022), teleost fishes are the largest group of extant vertebrates, accounting for nearly half of the species in the subphylum Vertebrata (Volff, 2005). However, only a few species from this large biodiversity pool are established animal models for investigating health and disease (Schartl, 2014; Lleras-Forero et al., 2020; Beck et al., 2022; Dohi and Matsui, 2022), evolutionary and developmental biology (Braasch et al., 2015; Gross and Powers, 2020), ecology, and the environment (Burnett et al., 2007; Barber, 2013; Bugel et al., 2014; Silva Brito et al., 2022). Teleost animal models include the zebrafish (*Danio rerio*), three-spined stickleback (*Gasterosteus aculeatus)*, mummichog (*Fundulus heteroclitus*), blind Mexican cavefish *(Astyanax mexicanus)*, turquoise killifish *(Nothobranchius furzeri)*, and the Japanese rice fish *(Oryzias latipes)*, amongst others.

However, teleost fishes are not just useful tools for the advancement of biomedical, evolutionary, and ecological research. Different food security projections indicate that the global food demand will increase between 31% and 56% between 2000 and 2050 (Baldos and Hertel, 2014; van Dijk et al., 2021). In 2019, aquatic food contributed a substantial amount of the protein consumed globally: 7% of all protein and 17% of all animal protein (FAO, 2022). This underscores the important role that food fish, from capture or aquaculture, have in global food security. Thus, further understanding of host-microbe interactions in aquatic species, especially in aquaculture that now represents 56% of the total aquatic animal food production (FAO, 2022), could elucidate solutions for a more sustainable fish food production for the growing world population.

Studies of microbiomes from farmed and wild teleost species, including different body sites and environmental samples [reviewed by (Llewellyn et al., 2014; Kelly and Salinas, 2017; Legrand et al., 2020; Lopez Nadal et al., 2020; Perry et al., 2020)] have provided new insights into the microbial contribution to survival (Schmidt et al., 2017), growth (Li et al., 2013), immunity and pathogenesis (Boutin et al., 2013; Lowrey et al., 2015). Other studies have also illustrated the effect that rearing systems (Dehler et al., 2017; Vestrum et al., 2018; Tarnecki et al., 2019; Minich et al., 2020; Bugten et al., 2022), feeds and feeding regimes (Bakke et al., 2013; Gajardo et al., 2017; Parris et al., 2019; Salger et al., 2020), and other environmental factors have on the fish-associated microbiota (Llewellyn et al., 2016; Sylvain et al., 2016; Krotman et al., 2020). Therefore, the increasing evidence of complex host-microbe-environment interactions driving the piscine host health warrants the development of gnotobiotic systems for their study. Since the first teleost gnotobiotic model of platyfish (*Xiphophorus maculatus)* was developed in 1942 (Baker et al., 1942), the number of teleost gnotobiotic systems remains limited, especially regarding fishes of economic and food security import. In spite of the numerous challenges that the generation and maintenance of gnotobiotic animals present, gnotobiotic systems for established animal models such as zebrafish (*Danio rerio*) or the three-spined stickleback (*Gasterosteus aculeatus)* have recently emerged (Pham et al., 2008; Milligan-Myhre et al., 2016; Lescak and Milligan-Myhre, 2017; Melancon et al., 2017; Zhang et al., 2020). Also, there are gnotobiotic experimental systems for food fish; including Atlantic halibut [*Hippoglossus hippoglossus*, (Verner-Jeffreys et al., 2003)], Nile tilapia [*Oreochromis niloticus*, (Situmorang et al., 2014)], Atlantic cod [*Gadus morhua*, (Forberg et al., 2011)], Dover sole [*Solea solea*, (De Swaef et al., 2017)], sea bass [*Dicentrarchus labrax*, (Dierckens et al., 2009)], and diverse salmonid species (Trust, 1974; Lesel and Lesel, 1976; Perez-Pascual et al., 2021). However, of the twenty species that account for 83.6% of the global aquaculture production (FAO, 2020), only Nile tilapia (Situmorang et al., 2014) has a reported gnotobiotic experimental system.

Atlantic salmon (*Salmo salar* L.) is one of the most important species in aquaculture. Atlantic salmon farming represented approximately 4.5% of the world’s aquaculture production in 2018, a global revenue of 15.4 billion USD, and 17.5 million meals (ISFA, 2018; FAO, 2020), highlighting the economic and food security significance of this species. Atlantic salmon is an anadromous species with a complex life cycle (Hoar, 1988; Klemetsen et al., 2003; Mobley et al., 2021). Early development in Atlantic salmon yolk sac fry is a temperature-dependent process, from fertilization to the initiation of external feeding (Gunnes, 1979; Gorodilov, 1996). During the yolk sac stage, the embryo utilizes maternal factors and nutrients deposited in the yolk sac for their growth and the ontogeny of tissues and organs (Sahlmann et al., 2015; Bizuayehu et al., 2019). Moreover, Atlantic salmon fry are associated with distinct microbial populations during this early stage of development (Lokesh et al., 2019).

Here, we developed a gnotobiotic experimental system for Atlantic salmon yolk sac fry. First, we screened a suite of chemicals for the external disinfection of fertilized eggs. Next, we established a derivation procedure to generate germ-free yolk sac fry from farmed and wild strains, and evaluated its potential adverse effects on the skin mucus barrier function. We further optimized the gnotobiotic husbandry to maintain fry throughout the yolk sac stage for 13 weeks post-hatching (wpH) at 6.5°C before first feeding. Finally, we used this gnotobiotic experimental system to investigate the microbial influence on the mucus barrier function, skin mucosa morphology, yolk consumption, body size, and adipose tissue accumulation during this early life stage.

## 2 Materials and methods

### 2.1 Animals

Fertilized farmed Atlantic salmon eggs (*Salmo salar* L., strain Aquagen) were provided by Aquagen AS (Trondheim, Norway). Fertilized wild Atlantic salmon eggs (*Salmo salar* L., strain Rauma) were provided by Haukvik Kraft-Smolt AS (Vinjeøra, Norway). Eggs were dispatched from their respective producers in compliance with their protocols between 349.18 and 414.10 degree-days for the farmed, and at 281 degree-days for the wild Atlantic salmon. All procedures were performed at fish room temperature (FRT) of 6.5°C. Fish experiments were conducted on yolk sac fry prior to exogenous feeding, and according to the Norwegian Animal Welfare Act (2010) and the EU directive on the Protection of Animals Used for Scientific Purposes (Directive 2010/63/EU). To euthanize yolk sac fry, MS-222 (E10521, Sigma Aldrich) was prepared as a stock solution of 0.52% (w/v) in salmon gnotobiotic media (SGM) buffered with 1M Tris pH 9 to a final pH of 7.5, and used at a final concentration of 520 mg/l. Death was confirmed by heartbeat cessation under a stereomicroscope.

### 2.2 Salmon gnotobiotic media, antibiotic cocktail, and chemical disinfectants

Sterile (autoclaved) salmon gnotobiotic media (SGM) was used for the rearing of Atlantic salmon yolk sac fry in all our experiments [96 mg/l NaHCO_3_, 60 mg/l CaSO_4_•2H_2_O, 60 mg/l MgSO_4_•7H_2_O, 4 mg/l KCl; (US EPA, 2002)]. SGM was prepared from 100X stocks of MgSO_4_•7H_2_O, KCl, and NaHCO_3_; and a 5X stock of CaSO_4_•2H_2_O. All stock solutions were autoclaved except NaHCO_3_, which was filter sterilized. After adding the appropriate volume of the stock salt solutions to Milli-Q water, SGM was sterilized by autoclaving in glass bottles. The antibiotic cocktail contained rifampicin, kanamycin, penicillin, ampicillin, oxolinic acid, erythromycin, and Amphotericin B (**Supplementary Table 1**). The cocktail was prepared by adding antibiotic stock solutions to sterile SGM, followed by filter-sterilization through a 0.22 µm PES membrane (431098, Corning), before aliquoting using sterile technique, and storage at -20° C until use. Bronopol, formaldehyde, glutaraldehyde, sodium hypochlorite, hydrogen peroxide, and Buffodine were used to prepare the disinfection solutions in SGM that were tested to establish the derivation procedure. Working concentrations of the chemical disinfectants, contact time, and references for their previous use in other freshwater, saltwater, and brackish water teleost species are listed in **Supplementary Table 1**.

### 2.3 Derivation of germ-free Atlantic salmon yolk sac fry

To generate germ-free (axenic) Atlantic salmon yolk sac fry, we externally disinfected fertilized eggs by immersion in an antibiotic cocktail, and subsequently in an iodine-based solution (i.e. derivation). Upon receipt from the producer, eggs were transferred to 14 mm ventilated Petri dishes containing sterile SGM at a density of 1 egg/ml and maintained at FRT for 24 hours for acclimation. After acclimation, the SGM in dishes assigned to germ-free (GF) yolk sac fry was exchanged for the antibiotic cocktail and incubated for 24 hours. In dishes assigned to non-disinfected yolk sac fry (conventionally raised, CVR), the SGM was exchanged for fresh sterile SGM. The derivation of GF yolk sac fry was conducted inside a laminar flow cabinet, using sterile technique, and sterile materials wiped with 70% ethanol and irradiated with UV light (except when photodegradable). Between 15 and 20 eggs were transferred to a sterile conical vial (62.547.254, Sarstedt) using single-use sterile plastic forceps (232-0191, VWR). The fertilized eggs were incubated in 50 ml of a Buffodine solution (50 mg/ml free iodine in sterile SGM) for 30 minutes. The conical vial was maintained horizontally to maximize the area of contact between the egg chorions and the solution, and it was gently mixed by rolling five times every ten minutes. After incubation, the eggs were rinsed four times in 50 ml of sterile SGM. The same rinsing and transfer procedure was used in the chemical screen for external disinfectants after derivation using the concentrations and contact times listed in **Supplementary Table 1**. CVR eggs were transferred from the 14 mm Petri dishes to the tissue culture flask directly. After removing the SGM from the last rinse, the eggs were decanted into a tissue culture flask (734-2788, VWR) containing 100 ml of sterile SGM and maintained as described in the next section.

### 2.4 Salmon gnotobiotic husbandry

#### 2.4.1 Maintenance of flasks containing yolk sac fry

All procedures after the derivation of germ-free fertilized eggs were conducted inside a laminar flow cabinet, using sterile technique, and sterile materials wiped with 70% ethanol, and irradiated with UV light (except when photodegradable or containing live animals). Eggs and hatched yolk sac fry were kept in tissue culture flasks with 100 ml sterile SGM in the upright position at FRT, in the dark. 60% of the SGM volume in the flasks was exchanged with sterile SGM three times a week to maintain water quality levels.

#### 2.4.2 Stocking density

A density of 15-18 yolk sac fry per 100 ml SGM was maintained in flasks until 10 weeks post-hatching (wpH). After 10 wpH, the fish density was reduced to ~8 fish/100 ml SGM to keep pace with fish growth and to prevent the decline in the water quality, since stocking density affects growth and other physiological traits in Atlantic salmon (Refstie and Kittelsen, 1976; Oppedal et al., 2011). Similar fish densities were maintained across replicate flasks in all the experiments.

#### 2.4.3 Conventionalization procedure

Previous work in other teleost fishes has shown that colonization of the host by the environmental microbiota occurs soon after hatching (Bates et al., 2006; Kanther and Rawls, 2010). Consequently, conventionally raised (CVR) yolk sac fry hatch from eggs that are not externally disinfected (i.e. not derived) and therefore are most likely colonized by microbes from the hatchery and transportation, whereas GF fry hatch from externally disinfected eggs (i.e. derived). The GF status was assessed for each experimental flask by culture and non-culture-based methods (see corresponding subsection). Conventionalized (CVZ) yolk sac fry are GF fry that were re-colonized by the addition of untreated fresh water from lake Jonsvatnet (63°22’14.9”N 10°35’30.9”E), collected at the Vikelvdalen water treatment plant (VIVA). Jonsvatnet lake water has a pH of 6.8. For conventionalization (the procedure to generate CVZ fry), GF flasks received 60 ml of Jonsvatnet water during the water exchange one week after experimental hatching day (when 60% of the eggs were hatched in all replicate flasks and across all treatments). CVR and GF flasks received 60 ml of autoclaved Jonsvatnet water. Both the Jonsvatnet untreated water and autoclaved water were equilibrated to fish room temperature overnight before they were added to the flasks.

#### 2.4.4 Water quality

Water quality parameters were measured in the SGM removed after the water exchange. Nitrate, nitrite, and total ammonia nitrogen (TAN) were measured using test kits (147008, 2745425, and 224100, respectively; HACH). pH was measured with a pH meter (MP220 Mettler Toledo). The dissolved oxygen was measured with an optical IDS dissolved oxygen sensor (FDO 925, Xylem Analytics). All measurements were conducted according to the manufacturer’s instructions. Water quality parameters were measured at 3 wpH, 6 wpH, and 9 wpH by sampling 4 flasks per microbial status (CVR, CVZ, and GF); and at 12 wpH in every flask (CVZ, n=8; CVR=8; and GF, n=10). The pH in the flasks ranged between 7 and 8, nitrite and nitrate remained at 0 ppm, and TAN between 0.02 and 2.8 mg/l (all microbial conditions, flasks, and timepoints included). Overall, the average water quality parameters measured in each microbial condition and timepoint were within optimal ranges recommended for salmon hatcheries: pH 6.2 to 7.8; nitrite and nitrate < 0.1 mg/l; TAN < 2.0 mg/l; oxygen > 80% (Hjeltnes et al., 2012). The only exception was at 9 and 12 wpH when the average TAN values per condition ranged between 2.1 and 2.5 mg/l for all microbial conditions. However, we remeasured these parameters after the water exchange and the TAN values dropped to <1.6 mg/l. The percent saturation of dissolved oxygen was measured at 12 wpH before and after the water exchange. Before the water exchange, the average saturation of dissolved oxygen was 62%, 61.3%, and 61.8%; for GF, CVR, and CVZ, respectively. However, the dissolved oxygen increased to 84.4%, 83,6%, and 84.7%; for GF, CVR, and CVZ, respectively; after the water exchange. These values were very similar to the dissolved oxygen measured in the autoclaved SGM used for the water exchange (85.8%).

#### 2.4.5 Culture-based sterility tests

The timing of sampling, the sample type, culture conditions, and types of growth media are listed in the Results and/or **Supplementary Material** sections for the corresponding experiments. Routinely, aerobic culture-based sterility tests of SGM from individual GF flasks were conducted on experimental hatching day to remove contaminated flasks. Aerobic culture-based sterility tests of SGM from individual fish flasks were conducted one week before the sampling of animals, after the sampling of animals, and/or at the end of each experiment. In addition, anaerobic cultured-based sterility tests of SGM from individual fish flasks were conducted at the end of the yolk-sac stage using the Anaerocult A system (113829, Millipore), according to the manufacturer’s instructions. Aerobic culture-based sterility tests of homogenates from individual single whole fry were also conducted at the end of the yolk-sac stage. After euthanasia with filter-sterilized MS-222, individual whole larvae were transferred to a sterile tube with screw cap, prefilled with 1.4 mm zirconia beads (432-0356, VWR), for homogenization using a Vortex Genie (SI0236, Scientific Industries) at maximum speed for five minutes. The following liquid media were used for the culture-based sterility tests and were prepared according to manufacturers’ instructions unless otherwise indicated: tryptic soy broth (TSB; 84675, VWR), Saboraud-2% Dextrose broth (SDB; 08339, VWR), nutrient broth (NB; 05443, Millipore), brain heart broth (BHIB; 10493, Millipore), and glucose yeast extract broth (GYEB; 10 g/l glucose, 2.5 g/l yeast extract). Tryptic soy agar (TSA) and glucose yeast agar (GYEA) were prepared using 15 g/l agar (20767.298, VWR) and TSB and GYEB as a base. 100 µl of SGM or of the whole fry homogenate were used as inoculum in 3 ml of liquid media, or directly on the surface of an agar plate without streaking. Growth media were incubated for up to one month post-inoculation, and inspected after 48 h, weekly, and at one-month post-inoculation. Uninoculated growth media and growth media inoculated with sterile SGM were used as negative controls. SGM from CVR or CVZ flasks was used as positive control. When sterility tests were performed on fry homogenates, the following controls were included: CVR fry homogenate, sterile zirconia beads and SGM used for homogenization, and the filtered sterilized MS-222 used for euthanasia.

#### 2.4.6 Flow cytometry

Flow cytometry analysis was conducted to determine bacterial cell counts in SGM from individual fish flasks to complement and confirm culture-based sterility tests. SGM was transferred to a sterile conical vial, and stored at FRT until analysis. The samples were diluted 1:10 in filter-sterilized 0.1X TE buffer (0.2 µm syringe filter with a SFCA membrane; 17823 K, Sartorius). To detect bacterial cells, samples were stained with dsDNA Nucleic Acid Stain SYBR™ Green I (S7563, Life Technologies Corporation) and incubated at room temperature for 15 minutes in the dark. The analysis was performed on a BD Accuri C6 Flow Cytometer (653118, BD Bioscience). This flow cytometer is equipped with two lasers (14.7 mW 640nm Diode Red Laser and 20 mW 488nm Solid State Blue laser), four fluorescence filters (533/30 nm, 585/40 nm, >670 nm, and 675/25 nm), and two scatter detectors (90° ± 13 and 0° ± 13). Validation of the flow cytometer was conducted daily with Spherotech 6-Peak Validation Beads for FL4 (653145, BD Bioscience) and Spherotech 8-Peak Validation Beads for FL1 - FL3 (653144, BD Bioscience). Samples were run for 1-2 minutes or until 20,000 events were reached on medium flow (35 µl/min). The threshold was set to channel 1000 on the FL1 signal. Positive controls from colonized CVZ and CVR flasks were used to distinguish bacteria from background particles. Negative controls from sterile SGM and filter-sterilized TE buffer were also included in the analysis. Samples were processed in the BD Accuri™ C6 Software. Green fluorescence (FL1, 533/30 nm) and red fluorescence (FL3, >670 nm) were used to distinguish the bacterial fluorescent signal from the background signal (inorganic and organic particles). Gates were manually drawn based on data obtained from the negative and positive controls. All samples were analyzed with the same gates. Statistics obtained from the software were exported to Microsoft Excel for further analysis.

### 2.5 Experimental design and statistical analyses

Unless indicated, the number of replicate flasks and/or of sampled fry for each experiment are indicated in the corresponding figures, figure legends, and/or **Supplementary Material**. The statistical tests applied for the multiple comparisons are indicated in each figure legend. When significant, the multiplicity adjusted *p*-value is reported for the comparison in the figure and text. All statistical tests were performed using GraphPad Prism version 9.0.0 for Windows, GraphPad Software, San Diego, California USA, www.graphpad.com.

### 2.6 Determination of excluded distance between fluorescent beads in mucus layer and the skin surface of yolk sac fry

**Figure 1:**
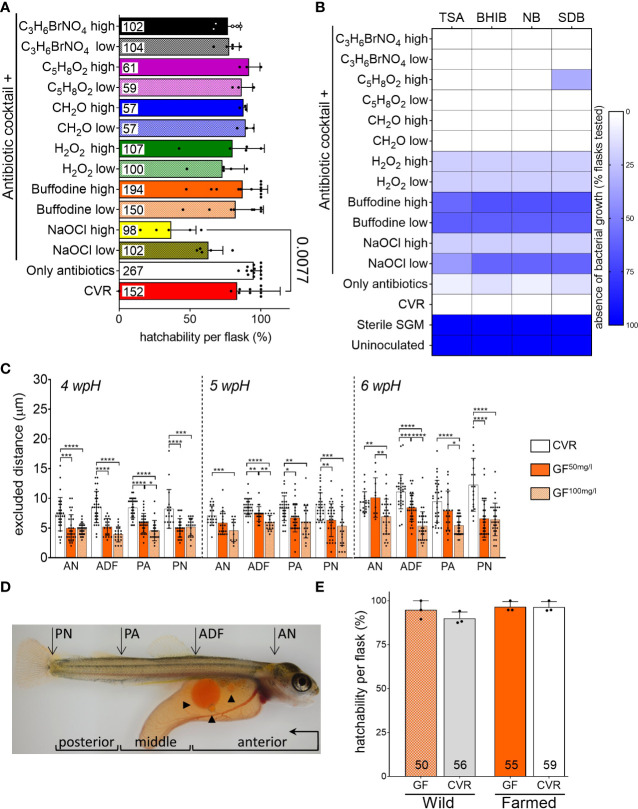
Effect of different derivation procedures on hatchability, microbial growth, and skin mucus layer of Atlantic salmon yolk sac fry **(A)** Hatchability per replicate flask two weeks after the expected hatching date (2 wpH), and after each derivation procedure. Chemical compounds used to disinfect eggs are designated by their molecular formula. Specific concentrations labeled as high and low in the graph, as well as the composition of the antibiotic cocktail are specified in **Supplementary Table 1**. Data shown was pooled from three separate derivations, each with a different batch of eggs. Bar graphs represent mean hatchability per treatment ± SD and the total number of eggs is reported at the bottom of each bar. Means per treatment were compared to non-disinfected (conventionally raised, CVR) controls using Kruskal-Wallis and the *post-hoc* Dunn’s multiple comparison tests. **(B)** Heatmap representing the results of culture-based sterility tests using salmon gnotobiotic media (SGM) sampled from flasks in **(A)** as inoculum. The color scale represents the percentage of replicate flasks per treatment where microbial growth was absent. Number of flasks per derivation procedure and culture media used are shown in **Supplementary Table 2**. Columns represent the culture media: TSA, tryptic soy agar; BHIB, brain heart infusion broth; NB, nutrient broth; and SDB, Saboraud-Dextrose broth. Samples were incubated aerobically at room temperature. SGM from CVR flasks was used as positive control; uninoculated media and sterile SGM, as negative controls. **(C)** Estimated functional thickness of the mucus-like barrier at 4 wpH, 5 wpH, and 6 wpH. Excluded distance between skin epithelial surface and 1 µm particles was measured in CVR and germ-free (GF) fry obtained using two different derivation procedures: antibiotic cocktail plus iodine at 50 mg/l or 100 mg/l (GF^50mg/l^ and GF^100mg/l^). Measurements were taken at the four body sites: AN, anterior notochord; ADF, anterior to the dorsal fin; PA, post-anal; and PN, posterior notochord. Bar graphs represent the mean excluded distance measured per bead ± SD. When possible, ten beads were measured per fry. Fry were raised in triplicate flasks per condition. One fry was sampled from each replicate flask, thus a total of two to three fry per condition were measured. Means were compared to each other using one-way ANOVA and *post-hoc* Tukey’s multiple comparison tests. Because of the number of comparisons, *p-*values are designated with asterisks (* for *p* ≤ 0.05, ** for *p* ≤ 0.01, *** for *p* ≤ 0.001, and **** for *p* ≤ 0.0001). **(D)** Representative micrograph of a 6 wpH CVR fry. Body sites (AN, ADF, PA, and PN) where the excluded distance was measured are indicated with arrows. Arrowheads point to some of the multiple oil globules in the yolk. Body segments sectioned for histology analysis are indicated with brackets: anterior (from the snout to the anterior of the dorsal fin), middle (from the anterior of the dorsal fin to the anal pore), and posterior (from the anal pore to the posterior end of the notochord). The direction of sectioning is indicated in the anterior segment bracket. **(E)** Hatchability per replicate flask at 2 wpH after derivation of wild and farmed eggs. Bar graphs represent mean hatchability per treatment ± SD and the total number of eggs is reported at the bottom of each bar. Means were compared to each other using Kruskal-Wallis and *post-hoc* Dunn’s multiple comparison tests.

Two sizes of carboxylate modified (charged, mucoadherent) fluorescent beads (FluoSpheres; F8823, F8801; Molecular Probes) were utilized in this study as described in Lai et al., 2007: 100 nm diameter red fluorescent (excitation maximum 580 nm; emission maximum 605 nm) and 1 µm diameter yellow-green fluorescent (excitation maximum 505 nm, emission maximum 515 nm). Fluorescent beads were maintained in an aqueous suspension containing 2% solids w/v in 2 mM sodium azide. After vortexing to prevent sedimentation, an aliquot of this suspension was added to sterile SGM to yield a final concentration of 0.002% w/v. Immediately after euthanasia, yolk sac fry were transferred to 3 ml fluorescent particle suspension in SGM and incubated for 10 minutes, rinsed by dipping in sterile SGM, and transferred to an imaging chamber with a 180 µm polymer coverslip base and locking lid (µ-Dish, Ibidi, Gräfelfing, Germany). Images were acquired using a Leica TCS SP8 microscope (Leica Microsystems, Wetzlar, Germany) using a 10x HC PL Apo CS objective with a working distance of 2.2 mm. The sample was illuminated with a white light laser with excitation lines at 505 nm for the yellow-green beads, at 580 nm for red fluorescent beads, and at 495 nm for reflectance imaging. Images were collected by 4 detector channels: reflectance (488-504 nm), yellow-green fluorescent (511-521 nm), red fluorescent (600-610 nm), and transmitted light to aid in the positioning of the sample. Z-stacked XY images (918x918 µm) were obtained from the surface of the coverslip to beyond the fish skin surface with a Z-step size of 0.5 µm at a scan speed of 8000 Hz, at four sites along the anterior-posterior axis of the yolk sac fry (**Figure 1D**). Single XY images were obtained at similar Z coordinates for comparison purposes. The excluded distance was defined as the Z distance between the maximum fluorescence signal from single 1 µm beads and the maximum reflectance signal from the skin surface at the same XY position. The stack profile intensity feature of the Leica LASX software was used to identify signal maxima for the bead fluorescence and skin reflectance signals in regions of interest located at the XY position of individual beads. Distances were obtained for a maximum of 10 beads per Z-stack per fish.

### 2.7 Growth and adipose tissue analysis using Nile Red

A cohort of 13 GF, 11 CVR, and 10 CVZ flasks was raised for the analysis of growth, adipose tissue, and survival between 3 and 13 wpH. Contaminated GF flasks were removed from the experiment (**Supplementary Table 3**). Quantification of adipose tissue deposition (total adipose tissue area) using Nile Red (N1142, Invitrogen) staining of neutral lipids was conducted as previously described in zebrafish (Minchin and Rawls, 2011; Tingaud-Sequeira et al., 2011; Minchin and Rawls, 2017a; Minchin and Rawls, 2017b) with a few modifications: 1) the staining solution was prepared in autoclaved SGM and fish were incubated for one hour at FRT; and 2) epinephrine was not used for the contraction of the melanosomes. After staining, fish were euthanized, and their whole gastrointestinal tracts were carefully dissected from esophagus to anus for imaging the associated adipose tissue. Only intact gastrointestinal tracts were imaged. Adipose tissue area, intestinal length after dissection, and standard length were measured for each fish, and only fish with the three measurements were analyzed. Fluorescence images of stained tissue and their corresponding brightfield images were acquired using a ZEISS Axio Zoom.V16 fluorescent stereo microscope (SYCOP/EMS3) equipped with a Plan-NeoFluar 1.0X objective, a HXP 200 C epi-fluorescence metal halide lamp, a 38HE filter set BP 470/40 excitation and BP 525/50 emission, and an AxioCam 506 camera (Carl Zeiss Microscopy GmbH). Fluorescent images were acquired at a constant exposure of 520 ms. ZEN 2012 software (Carl Zeiss Microscopy GmbH) was used for image acquisition. Morphometric characteristics were measured on lateral images of whole euthanized fry. We sampled two flasks at 3 wpH, 6 wpH, and 9 wpH, and 12 wpH (after splitting flasks). 10 to 14 fry were measured per experimental condition per timepoint except at 3 wpH, when due to a procedural error we could not measure area of the yolk and therefore only four fry were measured. The following morphometric characteristics (**Supplementary Figure 3A**) were measured on acquired images: standard length (SL, length from the tip of the snout to the end of the notochord, excluding caudal fin); height posterior to the dorsal fin (HPD, length from the posterior point of insertion of the dorsal fin to the ventral margin of the body, perpendicular to the SL axis); eye diameter (ED); and area of the yolk area (AY). Morphometric characteristics were measured for each fish, and only fish with all measurements were analyzed. Images were acquired using an Olympus SZX10 stereo microscope equipped with a DFPLAPO1x4 objective, and a SDC50 digital camera (Olympus). When the field of view of the camera could not capture the whole yolk sac fry, overlapping images were acquired. cellSense 2.2 software (Olympus) was used for image acquisition. All image analyses were conducted using FIJI/ImageJ 1.52p (Schindelin et al., 2012). Overlapping images were combined using the MosaicJ plugin (Thevenaz and Unser, 2007). All lengths were measured using the straight-line tool, except for the intestinal length, which was measured using the segmented line tool. The area of the yolk was manually traced using the polygon tool. The area of the intestinal adipose tissue was measured as described in (Minchin and Rawls, 2017b).

### 2.8 Histology

Single yolk sac fry were fixed in 15 ml of freshly made 4% paraformaldehyde in PBS (pH 7.4) for 48 h with very gentle rotation at FRT and stored at 4°C until processing. After fixation, fry were carefully deyolked and cut in segments along the anteroposterior axis using an industrial razor blade. Body segments were infiltrated stepwise overnight from 80% ethanol to paraffin using a TP1020 Semi-enclosed Benchtop Tissue Processor. The segments were laid horizontally with the same orientation along the dorsoventral axis and aligned at their anterior ends in a preliminary paraffin block, before the embedding in a final paraffin block that exposed the anterior end of all segments for sectioning. Ten 4 µm sections were obtained per fish per body segment. Mucosubstances were stained with Alcian Blue/PAS and nuclei with Mayer’s hemalum (101646, 101647, and 109249, respectively; Merck). Slides were mounted using NeoMount (109016, Merck). Images were acquired using a NanoZoomer SQ slide scanner (Hamamatsu) at 40X. NanoZoomer digital pathology for SQ 1.0.5 software (Hamamatsu) was used for image acquisition and export, as well as analyses including cell count and epithelial length measurement.

#### 2.8.1 Skin mucosa analysis at 4 wpH

The segments included: 1) posterior, from the tailfin to posterior of the anal pore; 2) middle, from the anal pore to anterior of the dorsal fin, and 3) anterior, from the dorsal fin to the snout; and are shown in **Figure 1D**. Six fry were randomly sampled for each experimental condition from two replicate GF flasks, and 3 replicate CVR and CVZ flasks. Serial sections were collected (anterior to posterior) from all three segments of individual fry. Four to nine sections, located at approximate equivalent positions, were imaged and quantified. All three body segments described above were examined.

#### 2.8.2 Adipose tissue verification at 12 wpH

The segments included: 1) posterior, from the tailfin to posterior of the anal pore, 2) mid-posterior, from the anal pore to anterior of the dorsal fin, 3) mid-anterior, from the anterior of the dorsal fin to anterior to the heart, and 4) anterior, from anterior to the heart to the snout; and are shown in **Supplementary Figure 3A**. Six fry were randomly sampled for each experimental condition from two replicate flasks. Serial sections were collected (anterior to posterior) from the mid-posterior segment of individual fry. Two to six sections were imaged to score the presence of adipose tissue per individual.

### 2.9 Analysis of skin and gut microbiota

Whole gastrointestinal tracts (gut) and skin microbiota were characterized by Illumina sequencing of 16S rRNA amplicons. Samples were collected from the cohort raised for the growth and adipose tissue analysis at 13 wpH. Three individuals from each of the two replicate CVR and CVZ flasks (24 samples in total) were sampled. To collect the tissues, individual yolk sac fry were transferred from the flasks into the first well of a row of a 12-well plate that was prefilled with sterile SGM. The SGM was removed and exchanged with filter-sterilized MS-222 for euthanasia. The fry were further rinsed by dipping in sterile SGM in the remaining wells in the row (total of three times) to minimize the contribution of bacteria from the aquatic environment of the original flasks to the skin microbiome analysis. After rinsing, individual fry were transferred to a sterile Petri dish bottom. The reminder liquid was removed by absorption to a Kimwipe without touching the sample, and by pipetting any excess of water. The fry was transferred to the Petri dish top for dissection using sterile technique under a stereoscope. After removing the yolk, the whole gastrointestinal tract was pulled from esophagus to anus using sterile forceps. Next, the yolk sac fry was decapitated, and the skin posterior to the gills dissected. We avoided dissecting the skin from the head as it could not be achieved reproducibly. Gut and skin from each individual fry were immediately transferred to separate sterile centrifuge tubes with screw caps, prefilled with 200 µl of 1.4 mm zirconia beads (432-0356, VWR) and TRIzol (15596026, Invitrogen). Beaded tubes containing 0.5 ml of TRIzol were used for gut samples and 1 ml TRIzol for skin samples. Samples were immediately frozen in liquid nitrogen and stored at –80°C until RNA extraction.

#### 2.9.1 RNA extraction and cDNA synthesis

Samples were homogenized in a Precellys 24 tissue homogenizer (Bertin Technologies) at 4000 rpm (skin samples) or 2500 rpm (gut samples) twice for 10 seconds. Total RNA was extracted using the Purelink RNA Mini Kit (Invitrogen™) following the manufacturer’s protocol. DNA was removed by using the On-Column Purelink DNase Treatment (Invitrogen) following the manufacturer’s protocol. Aliquots of the extracted RNA were immediately frozen at - 80°C. The concentration and quality of the RNA extract was assessed with a Nanodrop One Microvolume Spectrophotometer (Thermo Scientific™). Prior to cDNA synthesis, the RNA samples were diluted to 100 ng/µl, and a total amount of 800 ng was used for each cDNA synthesis reaction using the iScript cDNA Synthesis kit (Bio-Rad) following the manufacturer’s protocol.

#### 2.9.2 PCR amplification and amplicon library preparation

The broad coverage primers typically used to amplify the v3 + v4 region of the bacterial 16S rRNA gene have a high degree of similarity to *Salmo salar* rRNA sequences, resulting in co-amplification of fish sequences when using some of the previously published PCR primers. To avoid this, we designed a forward primer with low similarity to the *Salmo salar* 18S rRNA gene, but still showing good coverage for bacterial 16S rRNA gene sequences (Ill-329F: 5’-tcgtcggcagcgtcagatgtgtataagagacagnnnnACKGNCCWDACWCCTACGGG-3’, targeting sequence shown in capital letters). The coverage of the primer was evaluated using the Probematch tool of the Ribosomal Database Project (Cole et al., 2014). The 329F primer matches 11670 of the 12736 Type strain 16S rRNA gene sequences currently included in the RDP database, and as much as 12487 when one mismatch position is allowed. The primer Ill805R (5′-gtctcgtgggctcggagatgtgtataagagacag nnnnGACTACNVGGGTATCTAAKCC-3′) was used as reverse primer (Nordgård et al., 2017). PCR reactions (25 µl volume, 0.3 µM of each primer, 0.25 mM of each dNTP, 0.4 U, 1 µl cDNA as template) were conducted using Phusion Hot Start polymerase (Thermo Scientific). The following cycling conditions were used: an initial denaturation step at 98°C for 60 s; 38 cycles at 98°C for 15 s, 58°C for 20 s, and 72°C for 20 s; and a final elongation step of 5 minutes at 72°C. The resulting amplicons were purified and normalized using Sequal Prep™ Normalization plates (96 wells, Invitrogen). The amplicons were indexed in a second PCR using the Nextera^®^ XT Index Kit v2 Set A. The PCR conditions were as described above, except that 2.5 µl of the purified normalized PCR products were used as the template, together with 2.5 µl of each indexing primer. The same cycling program as described above, except for an annealing temperature of 50°C and only 10 cycles. After the indexing, the samples were purified and normalized using the Sequal Prep Normalization plate. Samples were pooled and concentrated using an Amicon^®^ Ultra 0.5 ml centrifugal filter (30K membrane, Merck Millipore), and analyzed with a NanoDrop™ One Microvolume Spectrophotometer (Thermo Scientific™). The amplicon library was sequenced in a MiSeq Illumina instrument with V3 reagents and 300 bp paired-end reads at the Norwegian Sequencing Center. The sequencing data was deposited at the European Nucleotide Archive (accession numbers ERS13490974 to ERS13490997).

#### 2.9.3 Data processing and multivariate statistics

The sequencing data were processed using the USEARCH pipeline v.11 (Edgar, 2010). The fastq_mergepairs command was used to merge sequence pairs, trim primer sequences, and filter merged sequences shorter than 390 bp. Quality-filtering was performed using the fastq_filter command with the default value of 1 for the expected error threshold. Amplicon sequencing variants (ASVs) were generated using the Unoise3 command (Edgar, 2016b) with the recommended minimum abundance threshold of 8 reads (in the whole data set). Taxonomy was assigned to the ASVs using the sintax command (Edgar, 2016a) and the Ribosomal Database Project (RDP) reference data set v18. The ASV table was normalized to 26 000 reads per sample. Statistical analyses were performed using the program package PAST (Hammer et al., 2001). Principal coordinate analysis (PCoA) was based on Bray-Curtis similarities (Bray and Curtis, 1957) and used to visualise differences in microbial community composition between samples. One-way PERMANOVA was used to test for significant compositional differences between groups (Anderson, 2001) based on Bray–Curtis similarities. Similarity percentage analysis (SIMPER) was used to identify the ASVs contributing most to the Bray-Curtis dissimilarities between sample groups (Clarke, 1993).

## 3 Results

### 3.1 Chemical screen for external disinfectants of Atlantic salmon eggs

We wanted to establish a derivation procedure to generate germ-free (GF) Atlantic salmon yolk sac fry (hereafter referred to as fry) by externally disinfecting fertilized eggs, and rearing them in sterile salmon gnotobiotic media (SGM, see Methods) until their hatching in a microbe-free aquatic environment. We first identified antibiotics and chemical disinfectants that effectively controlled microbial growth without affecting normal fry development. We tested thirteen different derivation procedures consisting of a 24 hour-incubation with an antibiotic cocktail alone, and in combination with a shorter exposure to chemical disinfectants previously used to generate GF fish from diverse teleost species (**Supplementary Table 1**). The disinfectants tested included bronopol (C_3_H_6_BrNO_4_), glutaraldehyde (C_5_H_8_O_2_), formaldehyde (CH_2_O), hydrogen peroxide (H_2_O_2_), sodium hypochlorite (NaOCl), and Buffodine; and were used at two different concentrations (**Supplementary Table 1**). We evaluated hatchability (percentage of hatched eggs per flask replicate) two weeks after the expected hatching date (2 wpH) to determine the effect of the derivation procedures on fry development. Mean hatchability across treatments ranged from 36.8% to 95% (**Figure 1A**). When comparing the hatchability of each treatment to conventionally raised (CVR) controls (i.e. non-disinfected eggs that remain associated with microbial communities from their conventional husbandry), the only treatment that significantly decreased hatchability in Atlantic salmon was the combination of the antibiotic cocktail with a 0.006% solution of sodium hypochlorite (NaOCl high, Kruskal-Wallis, Dunn’s multiple comparison test, *p* = 0.0077). This treatment resulted in 36.8% hatchability compared to 83.3% in the CVR flasks. Next, we tested the efficacy of these chemicals in controlling microbial growth after the derivation procedure. We sampled SGM from individual flasks at 2 wpH for inoculation of different culture media including tryptic soy agar (TSA), brain heart infusion (BHIB), nutrient broth (NB), and Saboraud-dextrose broth (SDB). Microbial growth was detected in all media inoculated with SGM from flasks housing CVR fry as early as 48 h after inoculation (**Figure 1B**, **Supplementary Table 2**). In contrast, no microbial growth was observed in media inoculated with sterile SGM or in the uninoculated control. The use of antibiotic cocktail by itself; or in combination with bronopol, formaldehyde, glutaraldehyde, or hydrogen peroxide all led to the absence of microbial growth in ≤33.3% of flasks housing fry in all media types tested. Also, the combination of antibiotic cocktail and 0.003% and 0.006% NaOCl solutions had a higher percentage of flasks (20% and ≤60%, respectively) with no microbial growth in each media tested (**Figure 1B**, **Supplementary Table 2**). However, the NaOCl solutions affected hatchability the most (**Figure 1A**). The combination of the antibiotic cocktail and Buffodine solutions containing 50 mg/l or 100 mg/l of free iodine were the most effective, as ≥58.3% and 62.5% percent of the flasks tested did not show microbial growth in each media tested, respectively (**Figure 1B**; **Supplementary Table 2**). Because these two iodine-based derivation procedures effectively controlled microbial growth without affecting hatchability, we selected them for further experiments.

### 3.2 Evaluation of potential adverse effects of the antibiotic and iodine-based derivation procedures for generating germ-free Atlantic salmon yolk sac fry

We further examined the potential adverse effects of the derivations procedures using the selected 50 and 100 mg/l solutions of free iodine (GF^50mg/l^ and GF^100mg/l^, respectively) in combination with the antibiotic cocktail. We investigated their effect on the skin, as this mucosal surface is the largest and outermost organ and is in direct contact with the environment. Confocal imaging of the skin mucosal surface of fry after immersion in an aqueous solution of mucoadherent fluorescent beads (100 nm and 1 µm in diameter) showed a clear separation between the location of the fluorescent signal from the beads and the reflectance signal from the skin epithelial surface. This indicates that neither the 100 nm nor the 1 µm fluorescent beads were able to access the skin epithelium, thus suggesting the presence of a functional mucus-like barrier. Next, we measured the distance between individual 1 µm mucoadherent fluorescent beads and the underlying skin epithelium (see Methods and **Supplementary Figure 1**). This distance, defined as excluded distance, provides a measurement of the functional thickness of the mucus-like barrier. We compared the excluded distance of CVR, GF^50mg/l^, and GF^100mg/l^ fry at 4 wpH, 5 wpH, and 6 wpH (**Figure 1C**). We examined four different body sites (**Figure 1D**) using the notochord (anterior end of the notochord, AN; posterior end of the notochord PN), the dorsal fin (anterior to the dorsal fin, ADF), and the anal pore (post-anal, PA) as reference points along the body axis of the fry. The excluded distance in GF^50mg/l^ fry was smaller (reduced mucus-like layer barrier function) than in CVR controls at all body sites and timepoints examined (one-way ANOVA, Tukey’s multiple comparison test, all *p* ≤ 0.05); except at the AN site at 5 and 6 wpH, and the PA site at 6 wpH (**Figure 1C**). In contrast, the excluded distance in GF^100mg/l^ fry was smaller than in CVR controls at all timepoints and all body sites measured (one-way ANOVA, Tukey’s multiple comparison test, all *p* ≤ 0.01; **Figure 1C**). Notably, the putative mucus-like layer appeared continuous, with no beads seen in contact with the skin surface in both derivation procedures. Furthermore, when comparing GF^50mg/l^ and GF^100mg/l^ fry, the excluded distance was significantly smaller in GF^100mg/l^ at the AN site at 6 wpH, at the ADF site at 5 and 6 wpH, and at the PA site at 4 and 6 wpH (one-way ANOVA, Tukey’s multiple comparison test, all *p* ≤ 0.05, **Figure 1C**). Thus, the excluded distance was significantly smaller in GF^100mg/l^ in at least one body site per timepoint. We also confirmed that hatchability in this experiment was not significantly altered by either derivation procedure, although hatchability in GF^100mg/l^ flasks was slightly lower (**Supplementary Figure 2A**). Finally, weekly sterility tests between 4 wpH and 6 wpH, using SGM samples from GF^50mg/l^ and GF^100mg/l^ flasks as inoculum, confirmed their microbial-free state (**Supplementary Figure 2B**). Together, these results show that while both derivation procedures generated germ-free fry, using the 100 mg/l free iodine may have an adverse effect on the skin mucosa. Accordingly, we selected the derivation procedure combining an antibiotic cocktail and a solution of Buffodine containing 50 mg/l of free iodine to generate GF fry for further experiments testing the robustness and applications of this novel method. Interestingly, these results may also suggest a microbial effect on the mucus barrier function since the excluded distance in GF fry was significantly smaller than in CVR fry at all timepoints and body sites (**Figure 1C**).

### 3.3 Derivation of fertilized eggs of a strain of wild Atlantic salmon

We next asked if our derivation procedure was also suitable to generate GF yolk sac fry from a wild strain of Atlantic salmon. We therefore derived eggs from one strain representing wild Atlantic salmon originating from the Rauma River (Haukvik GenBank AS), and the farmed Atlantic salmon used in the previous experiments (Aquagen AS). We evaluated the hatchability of GF and CVR eggs from both strains at 2 wpH. Mean hatchability across strains and treatments ranged from 90% to 100%, and there were no significant differences between the salmon strains or the microbial status (**Figure 1E**). In addition, our culture-based methods did not detect microbial contamination (**Supplementary Table 2**). Together, our results show that our derivation procedure is likely applicable to generate GF fry from different wild and domesticated Atlantic salmon strains.

### 3.4 Maintenance of gnotobiotic fry throughout the yolk sac life stage

**Figure 2:**
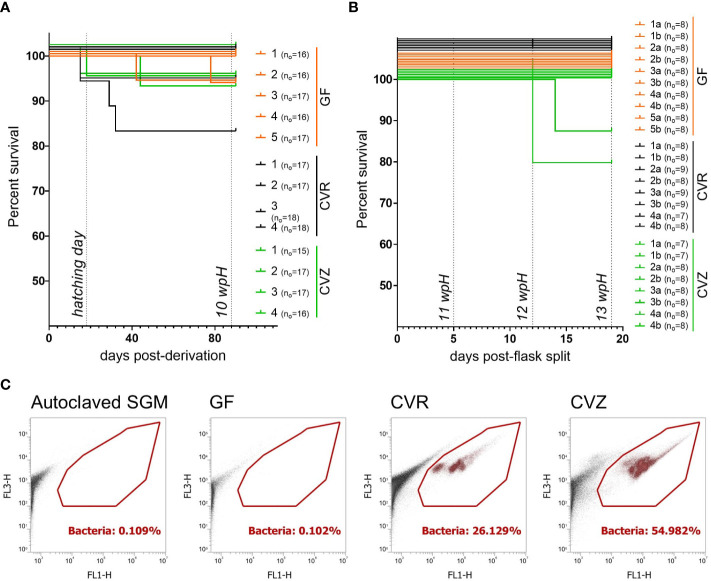
Gnotobiotic rearing of Atlantic salmon fry during the yolk sac stage **(A)** Survival curves from replicate flasks housing GF, CVR, and CVZ yolk sac fry until approximate 10 wpH. Days post-derivation and weeks post-hatching (wpH) are indicated on the x-axis. **(B)** Survival curves from replicate flasks housing GF, CVR, and CVZ yolk sac fry after splitting flasks and until 13 wpH. Days post-split and weeks post-hatching (wpH) are indicated on the x-axis. Survival curves were significantly different (p = 0.0129, Mantel-Cox and Gehan-Breslow-Wilcoxon tests). In **(A, B)** survival curves were compared using Mantel-Cox, Gehan-Breslow-Wilcoxon, and Log-rank tests. For visualization purposes only, survival curves were nudged on the y axis to separate overlapping curves. Initial number of fry (n_o_) per flask is indicated. **(C)** Representative plots from flow cytometry-based bacterial cell counts in SGM from GF, CVR, and CVZ flasks at 10 wpH. Each GF flask was analyzed at 10 wpH (before splitting flasks) and at 12 wpH. For the gating strategy for samples stained with SYBR™ Green I, Red/Orange fluorescence (FL3-H) versus green fluorescence (FL1-H) were plotted to distinguish the background from the bacteria (gated). The bacterial cell counts per flask and timepoint are shown in **Supplementary Table 4**.

Since we had only maintained GF and CVR fry for six weeks after hatching, we wanted to test if our gnotobiotic system, including the derivation procedure and gnotobiotic husbandry practices, was conducive to maintaining fry throughout the yolk sac stage. We generated a cohort of GF, CVR, and CVZ fry (i.e. GF fry that were recolonized with microbial populations from untreated water of a local lake after verification of their GF status; see Methods). We found no differences in the hatchability of flasks among the microbial conditions at 2 wpH, suggesting that colonization with different bacterial communities and/or the derivation procedure did not affect the development of this experimental cohort (**Supplementary Figure 2C**). Initially, we reared GF, CVR, and CVZ flasks for 72 days post-hatching (~10 wpH), with survival per flask ranging from 88.3% to 100% (**Figure 2A**). There were no significant differences between the survival curves from flasks assigned to the different microbial conditions (Mantel-Cox test, *p* = 0.3923; Gehan-Breslow-Wilcoxon, *p* = 0.3896). At 10 wpH, to prevent a decline in the water quality and to keep pace with the fry growth, we reduced the fish density in all experimental flasks, from an average of 16 fish to 8 fish, by splitting fish into two new flasks. In their new flasks, fish were maintained before ending the experiment prior to the full consumption of the yolk sac until 13 wpH. Only two of the 26 new flasks showed additional mortalities after the splitting (**Figure 2B**). Nevertheless, both flasks with less than 100% survival post-split (between ~10 wpH and 13 wpH) originated from the same flask (i.e. CVZ 4 in **Figure 2A**, split in CVZ 4a and 4b in **Figure 2B**). These results suggest that the difference in survival curves by 13 wpH was more likely a result of a flask effect rather than that of the conventionalization procedure.

We wanted to increase the stringency of our culture-based sterility tests as this cohort was reared for a longer period. Thus, we increased the number of culture media and conditions used to include TSA, SDB, NB, BHIB, glucose yeast extract agar (GYEA), and glucose yeast extract broth (GYEB) incubated aerobically at room temperature. In addition, TSA and GYEA were incubated aerobically at fish room temperature. We tested SGM samples from GF flasks at 3 wpH, 6 wpH, and 10 wpH. We found that all the flasks examined in **Figure 2A**, remained consistently germ-free during the ten-week period before the flask split under the culture conditions examined (**Supplementary Table 3**). Before ending the experiment at 13 wpH (**Figure 2B**), we duplicated the battery of culture media incubated at room temperature described above, to include anaerobic incubation using SGM samples as inoculum (**Supplementary Figure 2D**). We also sampled individual fry from GF flasks to conduct aerobic sterility tests of whole fry homogenates in GYEB, BHIB, NB, SDB aerobically; and in TSA, both aerobic- and anaerobically. No microbial growth was observed in any of the culture media, temperatures, or oxygen conditions tested at 12 wpH (**Supplementary Figure 2D**). Finally, we conducted a flow cytometry analysis to quantify the presence of unculturable bacterial cells in GF flasks. We compared bacterial cell counts in SGM from GF flasks; to CVR and CVZ flasks (positive controls), and autoclaved SGM (negative control). The bacterial cell counts (estimated by the number of gated events/µl) in SGM samples from GF flasks were equivalent to or less than the counts in freshly autoclaved SGM (0 to 10 counts, **Supplementary Table 4**). Notably, SGM from CVR and CVZ flasks had well-defined populations in the bacterial gate, that were not detected in SGM from GF flask at 10 wpH (**Figure 2C**; **Supplementary Table 4**), or at 12 wpH (**Supplementary Table 4**). Together, the results from culture- and non-culture-based sterility tests, as well as the survival analysis show that our derivation procedure and gnotobiotic husbandry practices are conducive to rearing GF, CVR, and CVZ fry throughout the yolk sac stage for 13 wpH at 6.5°C.

### 3.5 Effects of the microbial status on the functional thickness of the mucus layer and morphology of the skin mucosa of Atlantic salmon yolk sac fry

**Figure 3:**
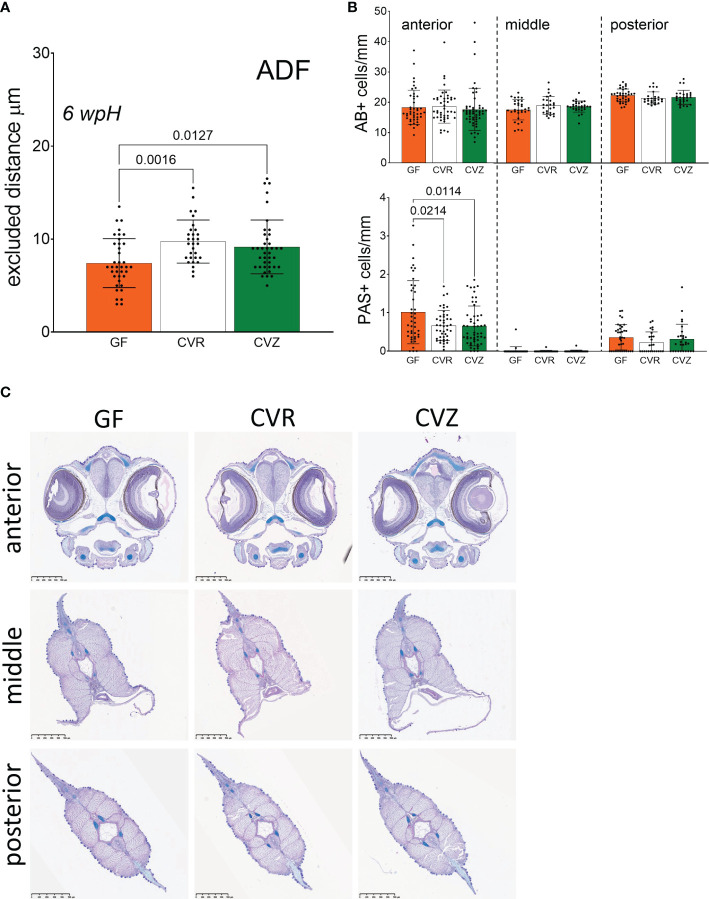
Use of the novel gnotobiotic system to study the microbial influence on the skin mucosa of Atlantic salmon yolk sac fry **(A)** Estimated functional thickness of the mucus-like barrier at 6 wpH. Excluded distance between skin epithelial surface and 1 µm particles was measured in GF, CVR, and conventionalized (CVZ) fry. Measurements were taken at the body site anterior to the dorsal fin (ADF). Bar graphs represent the mean excluded distance measured per bead ± SD. When possible, ten beads were measured per fry. Fry were raised in triplicate flasks per condition. Three to four fry were sampled from a single replicate flask, thus a total of three to four fry per condition were measured. Means were compared to each other using one-way ANOVA and *post-hoc* Tukey’s multiple comparison tests. **(B)** Mucus cells per mm of yolk sac fry skin enumerated at 4 wpH after staining with Alcian blue (AB+) and periodic acid-Schiff (PAS+). Fry were raised in triplicate flasks per condition. Two to three fry were sampled per replicate flask, thus a total of six fry per condition were processed for histology. Bar graphs represent the mean number of cells per mm of skin ± SD. Four to nine sections (see Methods) per fry were quantified per body segment (anterior, middle, and posterior, see Figure 1D). Means were compared to each other using one-way ANOVA and *post-hoc* Tukey’s multiple comparison tests. **(C)** Representative images of the stained sections corresponding to the three body segments examined and quantified in **(B)**.

Measurements of the functional thickness of the skin mucus-like layer showed differences between GF^50mg/l^, GF^100mg/l^, and CVR fry (**Figure 1C**). However, it remained unclear if it was a result of the iodine-based treatment during derivation, or of the germ-free status of the fry. To further study the chemical and microbial contributions to the thickness of the skin mucus-like layer, we generated a cohort of GF, CVR, and CVZ fry, and measured the excluded distance at 6 wpH at the ADF site. A comparison of the excluded distance among the three microbial conditions revealed that the functional thickness of the mucus-like barrier in both CVR and CVZ was larger than in GF flasks at the ADF site (one-way ANOVA, Tukey’s multiple comparison test, *p* = 0.0016 and *p* = 0.0127, respectively **Figure 3A**). These results indicate that the shorter excluded distance in GF fry is likely a result of the absence of microbes and not of an adverse effect of the derivation procedure used to generate GF fry, since CVZ fry underwent the same procedure before their re-colonization with microbial communities from a lake.

The localization of fluorescent beads relative to the skin epithelium indicated the presence of a mucus-like barrier covering the fry skin. We conducted a histological analysis to examine three body segments (anterior, middle, and posterior; **Figure 1D**) to confirm the presence of mucus-producing cells at this early life stage. We stained cells containing acidic (Alcian blue positive, AB+) and neutral (periodic acid–Schiff positive, PAS+) mucins in the skin, respectively. We found that fry skin at this stage does possess secretory cells with acidic mucosubstances (AB+), and fewer secretory cells with neutral mucosubstances (PAS+) in the skin mucosa at 4 wpH (**Figure 3B**). These cells are likely producing the mucus-like material that acted as a barrier between the mucoadherent fluorescent beads from the aquatic environment and the fry skin (**Figures 1C**, **3A**). We also evaluated if the microbial status affected the number of mucus cells in the skin of yolk sac fry by comparing CVR, CVZ, and GF fry at 4 wpH. There were no significant differences in the total number of AB+ mucus cells across the three microbial conditions at any of the body segments examined (**Figure 3C**). However, we found that the number of PAS+ mucus cells was lower in colonized (CVR and CVZ) than in GF fry in the anterior segment (one-way ANOVA, Tukey’s multiple comparison test, *p* = 0.0214 and *p* = 0.0114, respectively **Figure 3B**). Together, these results suggest that the presence of microbial communities in the aquatic environment may affect the morphology and function of the mucosal barrier in Atlantic salmon yolk sac fry at specific body sites.

### 3.6 Utilization of the gnotobiotic experimental system to study the microbial influence on growth, yolk consumption, and adipose tissue development

**Figure 4:**
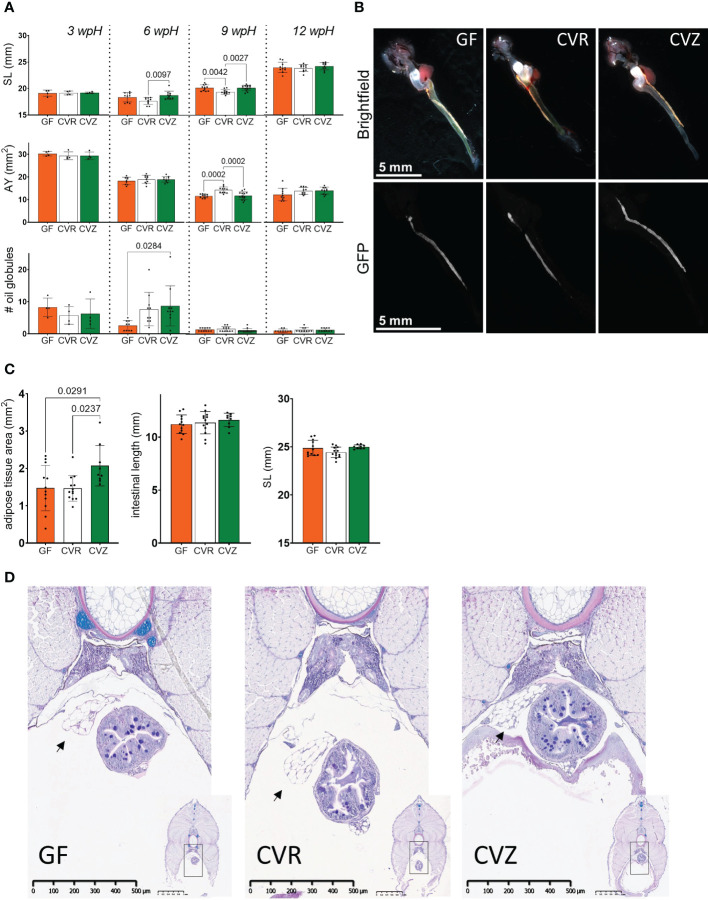
Use of the novel gnotobiotic system to study the microbial influence on Atlantic salmon yolk sac fry growth, yolk consumption, and adipose tissue accumulation **(A)** Measurements of standard length (SL), area of the yolk (AY), and number of oil globules from fry housed in GF, CVR, and CVZ flasks at 3 wpH, 6 wpH, 9 wpH, and 12 wpH. Two replicate flasks were sampled per microbial condition at each timepoint. Nine to fourteen fry were sampled per condition, except at 3 wpH (see Methods). The morphometric characteristics measured are illustrated in **Supplementary Figure 3A**. Bar graphs represent the mean of these measurements ± SD. Means were compared to each other using one-way ANOVA and post-hoc Tukey’s multiple comparison tests. **(B)** Representative images of dissected whole gastrointestinal tract, from the esophagus (posterior to gills) to the distal intestine of fry from GF, CVR, and CVZ flasks (top row, brightfield channel). Bottom row shows corresponding Nile red staining fluorescence images of adipose tissue associated with the intestine. Selected fry are representative of the mean intestinal length per treatment at 13 wpH. Difference in magnification was only used to display the entire length of the GI tract. **(C)** Measurements of the adipose tissue associated with the dissected intestine of fry from GF, CVR, and CVZ flasks at 13 wpH. Four to five replicate flasks were sampled, and a total of nine to thirteen fry per microbial condition. Corresponding measurements of the length of the intestine (measured posterior to the esophagus to the distal intestine) and SL are also included. Bar graphs represent the mean of these measurements ± SD. Means were compared to each other using one-way ANOVA and post-hoc Tukey’s multiple comparison tests. **(D)** Representative images of stained cross sections obtained at the mid-posterior body segment (see Methods and **Supplementary Figure 3A**) of GF, CVR, and CVZ fry at 12 wpH. Higher magnification images show the morphology of adipose tissue (arrows) adjacent to the intestine. Lower magnification images are included for orientation in the section, and the black box marks the approximate region of interest. Three fry were sampled per duplicate flask, thus a total of six fry per condition were processed for histology and examined to score the presence of adipose tissue associated to the intestine and other viscera.

We measured morphometric characteristics during the yolk sac stage at 3 wpH, 6 wpH, 9 wpH, and 12 wpH to interrogate the microbial influence on growth and development in Atlantic salmon yolk sac fry. We measured standard length (SL), height at posterior of the dorsal fin (HPD), and eye diameter (ED) (**Supplementary Figure 3A**). We compared GF, CVR, and CVZ flasks. CVR and CVZ fry are likely colonized by distinct microbial populations: in the case of CVR, by microbes from the hatchery and transport; whereas in the case of CVZ, by microbes from a freshwater lake. We did not observe significant differences in the SL of fry among the different microbial conditions examined at 3 wpH (**Figure 4A**). At 6 wpH, CVZ fry had a greater SL than fry from CVR flasks (one-way ANOVA, Tukey’s multiple comparison, *p* = 0.0097), but were not significantly different from fry in GF flasks. At 9 wpH, fry from CVR flasks had a smaller SL than fry from both GF and CVZ flasks (one-way ANOVA, Tukey’s multiple comparison, *p* = 0.0042 and *p* = 0.0027 respectively). However, no differences in SL across the different microbial conditions were further observed at 12 wpH, the timepoint closest to the end of the yolk sac stage examined (**Figure 4A**). HPD and ED measurements followed the same pattern as the SL, except that there was no difference in HPD among the microbial conditions at 6 wpH (**Supplementary Figure 3B**).

Since yolk sac fry rely on their yolk as a nutrient source for growth and development prior to exogenous feeding, we also investigated the effect of the microbial conditions on its utilization. We measured the two-dimensional area of the yolk (AY) from a lateral view to estimate its consumption. There were no differences in AY across the microbial conditions at 3 wpH and 6 wpH (**Figure 4A**). However, at 9 wpH the AY in fry from CVR flasks was larger than in GF and CVZ fry (one-way ANOVA, Tukey’s multiple comparison, *p* = 0.0002 for both comparisons). This suggests that the yolk was consumed more slowly in the CVR fry. Notably, these results mirrored those of the growth measurements at 9 wpH: CVZ fry with a smaller SL, HPD, and ED had consumed less of their yolk. At 12 wpH, similar to the other morphometric characteristics measured, no differences in AY were detected among the different microbial conditions. In addition, we observed a decrease in the number of oil globules in the yolk with the progression of the yolk sac stage in all microbial conditions (illustrated in **Figures 1D** and **Supplementary Figure 3A**). Notably, the only significant difference between the microbial conditions was observed at 6 wpH, when the number of oil globules was significantly larger in fry from CVZ flasks than in fry from GF flasks (one-way ANOVA, Tukey’s multiple comparison, *p* = 0.0284; **Figure 4A**).

Having observed a microbial influence on the use of yolk sac stores for growth, which likely includes the mobilization of lipids, we next asked if the microbial status also affected the accumulation of body fat. Nile Red staining, a technique previously used in zebrafish to measure growth and development in adipose tissue (Minchin and Rawls, 2011; Tingaud-Sequeira et al., 2011; Minchin and Rawls, 2017a; Minchin and Rawls, 2017b), revealed the presence of an adipose tissue (AT) depot associated with the intestine at the end of thirteen weeks of rearing (**Figure 4B**). A comparison among the microbial conditions showed that the size of the stained AT in CVZ fry was larger than in GF and CVR fry (one-way ANOVA, Tukey’s multiple comparison, *p* = 0.0291 and *p* = 0.0237 respectively; **Figure 4C**). Moreover, there were no significant differences in the length of the intestine or in SL of the sampled fry, suggesting that the difference in AT size was not a result of an overall difference in body size (**Figure 4C**). Together, these data suggest that microbial communities associated with fry from CVZ flasks may differentially promote the accumulation of adipose tissues in Atlantic salmon yolk sac fry. In addition to the live stained tissue, micrographs of cross sections obtained at the midposterior body segment (**Supplementary Figure 3A**) of fry at 12 wpH, also confirmed the presence of large unilocular cells clustered adjacent to the intestine and other viscera, in all fry sampled from GF, CVR, and CVZ flasks. The volume of these cells is largely occupied by a single vacuole surrounded by a thin layer of cytoplasm, a morphology consistent white adipocytes (**Figure 4D**).

**Figure 5:**
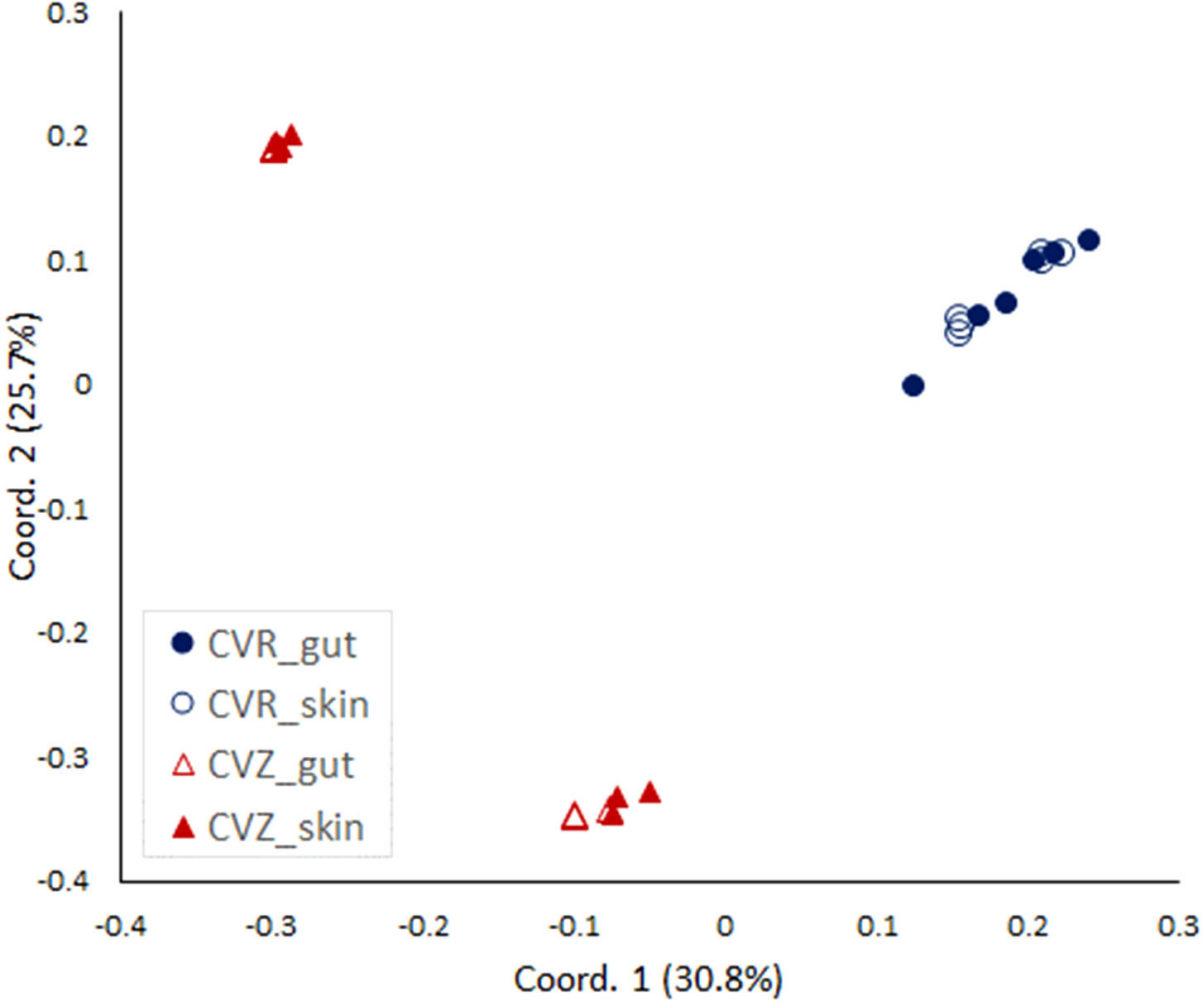
Analysis of bacterial communities in conventionally raised (CVR) and conventionalized (CVZ) flasks. PCoA plot based on Bray-Curtis similarities comparing gut and skin microbiota associated with fry from CVZ and CVR flasks at 13 wpH. Three individuals from two replicate flasks were sampled, and their whole gastrointestinal tract (gut) and skin were dissected. The v3+v4 16S rRNA amplicon sequencing was based on total RNA extracts that were DNase treated and reverse transcribed prior to the PCR amplification.

Finally, we wanted to confirm the putative difference in the composition of bacterial communities associated with fry reared in CVR and CVZ flasks. We used Illumina sequencing of RNA-based 16S rRNA amplicons to analyze bacteria associated with the whole intestinal tract (gut) and the skin dissected from 13 wpH fry reared in CVR and CVZ flasks, A PCoA ordination based on Bray Curtis distances showed that the skin and the gut microbiota of individual fry were highly similar in both CVR and CVZ flasks (**Figure 5**). This was supported by PERMANOVA analysis of gut and skin communities (gut vs. skin in CVR flasks, *p* = 0.1431; and gut vs. skin in CVZ flasks, *p* = 0.5697). However, we found a significant difference in the composition of bacterial communities between fry from CVR and CVZ flasks (skin and gut samples combined, PERMANOVA, *p* = 0.0001). Interestingly, CVZ samples clustered according to the rearing flask from which the fry were sampled, indicating a difference in the composition of bacterial communities between flasks (PERMANOVA *p* = 0.0026). Finally, we verified the bacterial density in SGM from CVR and CVZ flasks by CFU counts and did not find a significant difference. 

A SIMPER test was used to identify the ASVs contributing the most to the Bray-Curtis dissimilarity between the CVR and CVZ fry microbiota (**Supplementary Table 5**). ASV1 (classified as genus *Pseudomonas*) contributed the most to the Bray-Curtis dissimilarity (17.84%) and was highly abundant in the CVZ, but not in the CVR samples (mean relative abundances of 35.08% and 0.13%, respectively; **Supplementary Table 5**). Next, ASV12 (classified as family Comamonadacea) contributed 9.32% to the Bray-Curtis dissimilarity. This ASV was highly abundant in samples from CVR flasks, but very rare in the CVZ samples (on average 18.31% and 0.01% relative abundance, respectively; **Supplementary Table 5**). Interestingly, another member of family Comamonadacea, ASV16, contributed 6.59% to the Bray-Curtis dissimilarity. ASV16, classified as genus *Polaromonas*, was present in the CVZ samples (mean relative abundance of 12.96%; **Supplementary Table 5**) but was not observed in the CVR samples (**Supplementary Table 5**). Taken together, these results demonstrate that the bacterial communities associated with fry sampled from CVR and CVZ are distinct. In addition, these results suggest that this difference in composition may explain their also distinct adipose tissue accumulation phenotypes. 

## 4 Discussion

Here we present, to the best of our knowledge, the first gnotobiotic experimental system for Atlantic salmon (*Salmo salar* L.). We expected not only genetic and phenotypic differences between farmed and wild Atlantic salmon (Glover et al., 2017), but also differences in the microbial populations associated with their eggs and influenced by environmental and biogeographic factors (Llewellyn et al., 2016; Uren Webster et al., 2020). Therefore, we successfully tested the robustness of this experimental system in both a farmed and a separate wild Atlantic salmon strain. Notably, the overall success of the derivation procedure was relatively high (87.8% germ-free flasks by 2 wpH, 41 flasks examined), and was conducted using common laboratory equipment. Thus, we anticipate that this derivation procedure and husbandry practices can be replicated in other laboratories to routinely examine a large number of GF fry, hatched from eggs available year-round from commercial breeders. Also, we described in detail the development of the derivation procedure to obtain GF Atlantic salmon yolk sac fry to provide a framework for developing gnotobiotic experimental systems for other fishes.

Teleost gnotobiotic models offer several advantages over mammalian gnotobiotic models [reviewed in (Forberg and Milligan-Myhre, 2017; Zhang et al., 2020)]. We were able to maintain fry throughout the long yolk sac stage characteristic of this cold-water fish. Despite the conservation of developmental programs across vertebrates, the rate at which they are executed differs across species (Ebisuya and Briscoe, 2018; Rayon et al., 2020). For example, the process of somitogenesis, which in zebrafish takes ~25 minutes, takes 90 minutes in chicken, and five hours in humans (Hubaud and Pourquie, 2014). The distinct developmental time of Atlantic salmon may contribute a different time-scale resolution to the study of the microbial influence on development. In addition, when compared to other fishes, the large size of the Atlantic salmon yolk sac fry larvae is advantageous for the dissection of tissues and organs as well as for diverse imaging techniques, a characteristic on which we capitalized for the study of the skin mucus layer and the adipose tissue.

We used an innovative confocal microscopy approach, developed from the *in situ* mucus measurements of Atuma et al. (Atuma et al., 2001), to study adverse effects of chemical disinfectants on the skin mucosa at this early life stage. We showed that while the 50 mg/l and 100 mg/l free iodine-containing solutions of Buffodine did not significantly reduce hatchability [a common endpoint for toxicity tests in fish early life stages (OECD, 2013)], they did differentially affect the barrier function of the mucus layer. These results highlight the importance of identifying more subtle adverse effects that may act as confounding factors during experiments. It is worth noting that the 100 mg/l iodine-containing solution is equivalent to the suggested concentration for disinfecting eyed eggs at Atlantic salmon hatcheries. Whereas our derivation procedure used a longer contact time than at hatcheries our results may suggest that current practices in aquaculture disrupt the mucosal barrier in yolk sac fry during this vulnerable developmental stage. Maintenance of the mucus layer is critical for fishes because of its multiple functions (Reverter et al., 2018; Cabillon and Lazado, 2019) in behavior (Grutter et al., 2011; Satoh and Sowersby, 2021), immunity and barrier function (Benhamed et al., 2014; Salinas, 2015; Brinchmann, 2016; Tiralongo et al., 2020), locomotion (Zhang et al., 2022b), gas and osmotic exchange (Wright, 2021), amongst others. Moreover, an intact mucus layer is key for a teleost gnotobiotic model, as it is a niche for bacterial colonization (Weber et al., 2010; Gomez et al., 2013; Carda-Diéguez et al., 2017; Reinhart et al., 2019).

We measured the distance that mucoadherent particles of 1 µm can penetrate the thickness of the mucus layer (excluded distance), therefore estimating the functional thickness of the mucus layer relative to the particle’s characteristics. One limitation of this method is that we were constrained to a particle size that allowed us to accurately determine its position at the magnification used in this study. However, we do not exclude the possibility of using particles of different sizes and surface characteristics to map the physicochemical, and maybe even the biological “topography” of this fluid. The excluded distance method may reveal novel aspects of skin mucus dynamics. For example, we observed that the mucus layer was thinner in GF than in CVR fry at the AN site at 4 wpH, while histological methods showed that GF fry had an increased number of a specific type of mucus-secreting cells (PAS+), when compared to colonized fry (CVR and CVZ). For now, we can only speculate about the reasons why a microbially-induced increase in the number of mucus-secreting cells may precede a decreased mucus layer thickness. However, different mucins structures have somewhat different functions (Bonser and Erle, 2017), thus an increase in a particular mucin may not necessarily correlate with increased barrier function. Also, microbial colonization has been shown to alter glycosylation in intestinal mucins in the mouse (Johansson et al., 2015) and PAS staining is determined by the glyco-structures of the mucin. These preliminary results are only a snapshot of the microbial influence on the skin mucosa. Future work should include a detailed developmental analysis of the skin mucosa using these and other methods, to investigate the microbial influence on the morphology of the skin mucosa, as well as on mucus production dynamics and physicochemical properties in the context of normal yolk sac larvae development.

Our novel gnotobiotic experimental system can be a versatile tool to test the microbial contribution to diverse aspects of growth and development during this early life stage. In the present study, we found a transient microbial influence on body size and yolk consumption since by 12 wpH, closer to the complete resorption of the yolk, there were no significant differences in SL, AY, ED, or HPD. Interestingly, we noticed during the growth analysis, that in addition to the larger and more conspicuous oil globule in the yolk cell, there were other smaller ones visible at the magnification used to measure the SL of yolk sac fry. Moreover, we observed an increase in the number of these oil globules in fry from CVZ flasks, colonized with distinct microbial communities from a local lake. Changes in the size and number of oil globules are likely a consequence of changes in the metabolism of this lipid depot. Yolk lipids are stored as lipid moieties of vitellogenin; as well as in neutral lipid storage depots of oil droplets and globules (Silversand and Haux, 1995; Hiramatsu et al., 2015). In fact, the number, size, location, and use of oil globules varies greatly among fish species (Baras et al., 2018). Nevertheless, the mechanisms involved in the structure, catabolism, and biogenesis of oil globules in the teleost yolk cell, remain largely unknown compared to neutral lipid storage organelles in other cell types in plants and animals (Meyers et al., 2017; Onal et al., 2017; Thiam and Beller, 2017; Schott et al., 2019; Zhang et al., 2022a). Using Nile Red staining and histology analysis, we identified adipose tissue depots conspicuously associated with the intestine, and consisting of white adipocytes. This is consistent with previous studies in zebrafish that have shown that visceral adipose tissues are the first to develop (Flynn et al., 2009; Imrie and Sadler, 2010; Minchin and Rawls, 2017b). We found that CVZ fry had larger adipose depots than CVR or GF fry. Therefore, the presence of specific bacterial communities found in colonized CVZ but not in CVR fry, may affect both the mobilization of lipids from the maternal yolk depots (non-dietary), and the subsequent visceral adipose tissue storage during this early life stage. This phenotype could be advantageous during the first-feeding transition by providing a positive energy balance, preceding a period of starvation and more active swimming (China and Holzman, 2014; Voesenek et al., 2018), critical for larval survivorship of many aquaculture species (Holm, 1986; Garrido et al., 2015; Vadstein et al., 2018; Benini et al., 2022; Malzahn et al., 2022). In fact, previous work from others has shown that microbial colonization promotes adipose tissue accumulation and that specific microbial communities regulate adipose tissue accumulation (Bäckhed et al., 2004; Ridaura et al., 2013).

In the future, this gnotobiotic experimental system could be further exploited to manipulate early microbial exposures through the monoassociation with bacterial isolates or the colonization with complex microbial communities. Teleost gnotobiotic models have been successfully used for mechanistic studies of early colonization, assembly, succession, pathogen resistance, and probiotic colonization (Rawls et al., 2007; Rendueles et al., 2012; Wiles et al., 2016; Burns et al., 2017; Sundarraman et al., 2020; Stressmann et al., 2021). Importantly, early microbial experiences have a long-term influence on the life of humans and other vertebrates via colonization resistance, regulation of the immune development, and epigenetic DNA imprinting, amongst other mechanisms (Indrio et al., 2017; Torow and Hornef, 2017; Brodin, 2022). The long-term effect of early-life microbial experiences has also recently been explored in conventionally raised Atlantic salmon and Nile tilapia (Uren Webster et al., 2020; Deng et al., 2021). There remains a gap to bridge between the controlled manipulation of the microbial environment in gnotobiotic systems and commercial scale trials that would ultimately validate the long-term consequence of early bacterial exposures. This gnotobiotic experimental system is a step towards the integration of mechanistic and ecological insights to develop interventions at the farm level that will contribute to fish welfare and a more sustainable production of Atlantic salmon.

## Data availability statement

The datasets presented in this study can be found in online repositories. The names of the repository/repositories and accession number(s) can be found below: https://www.ebi.ac.uk/ena, ERS13490974 to ERS13490997.

## Ethics statement

Ethical review and approval was not required for the animal study because fish experiments were conducted on yolk sac fry prior to exogenous feeding. All experiments were still conducted in accordance with the Norwegian Animal Welfare Act (2010) and the EU directive on the Protection of Animals Used for Scientific Purposes (Directive 2010/63/EU).

## Author contributions

SG, CN, and IB conceived and designed experiments. SG wrote the original manuscript. CN, AM, IB, and ED contributed to sections of this manuscript. SG, CN, AM, and ED conducted experiments. SG, CN, AM, ED, and IB analyzed data. IB, OV and CN secured the funding. All authors contributed to the article and approved the submitted version.

## Supplementary material

Supplementary Figure 1 Schematic of a series of XY fluorescence and reflectance images obtained at regular intervals in the Z direction through the skin mucosa of Atlantic salmon yolk sac fry. The XYZ location of a mucoadherent bead in the mucus layer (green circle in plane a) was identified. The Z position of the skin reflectance signal (plane b) at the same XY coordinates was also identified. The difference in the Z position, defined as the excluded distance, estimates the extracellular barrier preventing the contact between the bead and the surface of the skin epithelium. a, XY plane in which the mucoadherent bead is located. a’, image of 555 nm fluorescence signal from 1 μm mucoadherent beads. b, XY plane of the reflectance signal from the skin relative to the XY position of the identified bead. b’, image of reflectance signal from the skin epithelium.

Supplementary Figure 2
**(A)** Hatchability per replicate flask at 2 wpH after two different derivation procedures (GF^50mg/l^ and GF^100mg/l^). Bar graphs represent mean hatchability per treatment ± SD and the total number of eggs is reported at the bottom of each bar. Means per derivation procedure were compared to non-disinfected (conventionally raised, CVR) controls using Kruskal-Wallis and the *post-hoc* Dunn’s multiple comparison tests. **(B)** Heatmap representing the results of culture-based sterility tests, using salmon gnotobiotic media (SGM) as inoculum, sampled from flasks in **(A)** at 4 wpH, 5 wpH, and 6 wpH. The color scale represents the percentage of replicate flasks per treatment where microbial growth was absent. Columns represent the culture media: TSA, tryptic soy agar; BHIB, brain heart infusion broth; NB, nutrient broth; and SDB, Saboraud-Dextrose broth. Samples were incubated aerobically at room temperature. SGM from CVR flasks was used as positive control; uninoculated media and sterile SGM, as negative controls. **(C)** Hatchability per replicate flask at of non-disinfected (conventionally raised, CVR), GF, and recolonized germ-free yolk sac fry (conventionalized, CVZ) at 2 wpH. Bar graphs represent mean hatchability per microbial condition ± SD, and the total number of eggs per treatment is reported at the bottom of each bar. Means per condition were compared to each other using Kruskal-Wallis and the *post-hoc* Dunn’s multiple comparison tests. **(D)** Graphical summary of results from sterility tests of SGM and fry homogenates sampled from GF flasks at 12 wpH. Columns correspond to individual flasks and controls; and rows, to the culture media and conditions used. Culture media, oxygen condition (-O_2_, anaerobic; +O_2_, aerobic), temperature (RT, room temperature) and the relevant controls per sample type are listed in the figure (also see Materials and Methods). Color representation of results is indicated in the legend.

Supplementary Figure 3
**(A)** Representative micrograph of a 12 wpH CVR fry used to illustrate the morphometric characteristics measured in the analysis conducted between 3 wpH and 12 wpH. Measurements included standard length (SL), height at posterior of dorsal fin (HPD), eye diameter (ED), and area of the yolk area (AY). Arrowhead points to the single large oil globule in the yolk. Body segments sectioned for histological analysis of adipose tissue are indicated with brackets: anterior, midanterior, midposterior, and posterior. The direction of sectioning for the histology analysis in the midposterior segment is indicated. **(B)** Measurements of ED and HPD from fry housed flasks in GF, CVR, and CVZ flasks at 3 wpH, 6 wpH, 9 wpH, and 12 wpH. Two replicate flask were sampled per condition each timepoint. Nine to fourteen fry were sampled per condition, except at 3 wpH (see Methods). Bar graphs represent the mean of these measurements ± SD. Means were compared to each other using one-way ANOVA and *post-hoc* Tukey’s multiple comparison tests.
