# Supplementary figures and images for: A novel gnotobiotic experimental system for Atlantic salmon (Salmo salar L.) reveals a microbial influence on mucosal barrier function and adipose tissue accumulation during the yolk sac stage
Source: Front Cell Infect Microbiol. 2023 Feb 1;12:1068302. doi: 10.3389/fcimb.2022.1068302 (PMC9929952; doi:10.3389/fcimb.2022.1068302)

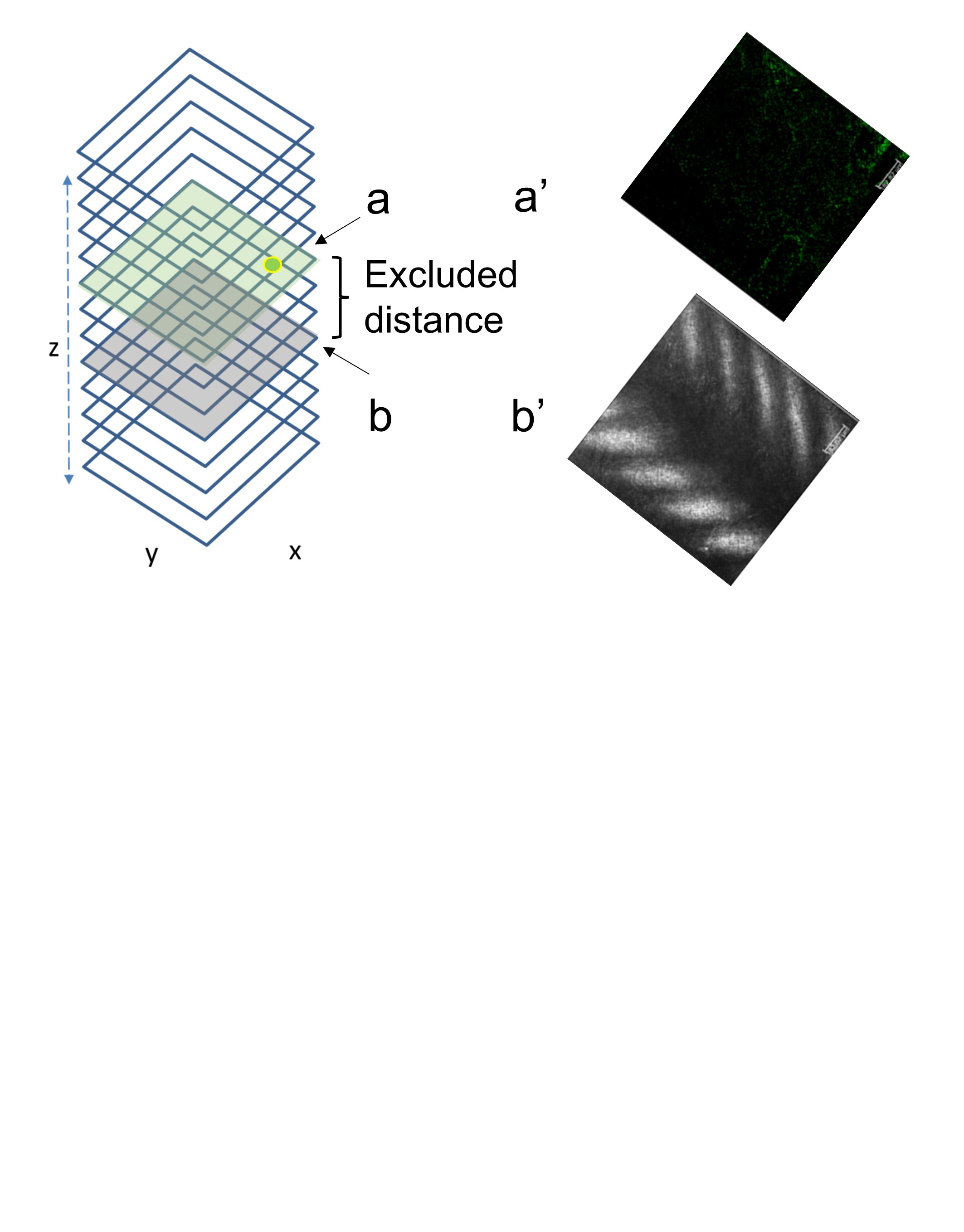

Supplement: Supplementary Figure 1 — Schematic of a series of XY fluorescence and reflectance images obtained at regular intervals in the Z direction through the skin mucosa of Atlantic salmon yolk sac fry. The XYZ location of a mucoadherent bead in the mucus layer (green circle in plane a) was identified. The Z position of the skin reflectance signal (plane b) at the same XY coordinates was also identified. The difference in the Z position, defined as the excluded distance, estimates the extracellular barrier preventing the contact between the bead and the surface of the skin epithelium. a, XY plane in which the mucoadherent bead is located. a’, image of 555 nm fluorescence signal from 1 μm mucoadherent beads. b, XY plane of the reflectance signal from the skin relative to the XY position of the identified bead. b’, image of reflectance signal from the skin epithelium. [file Image_1.jpeg]

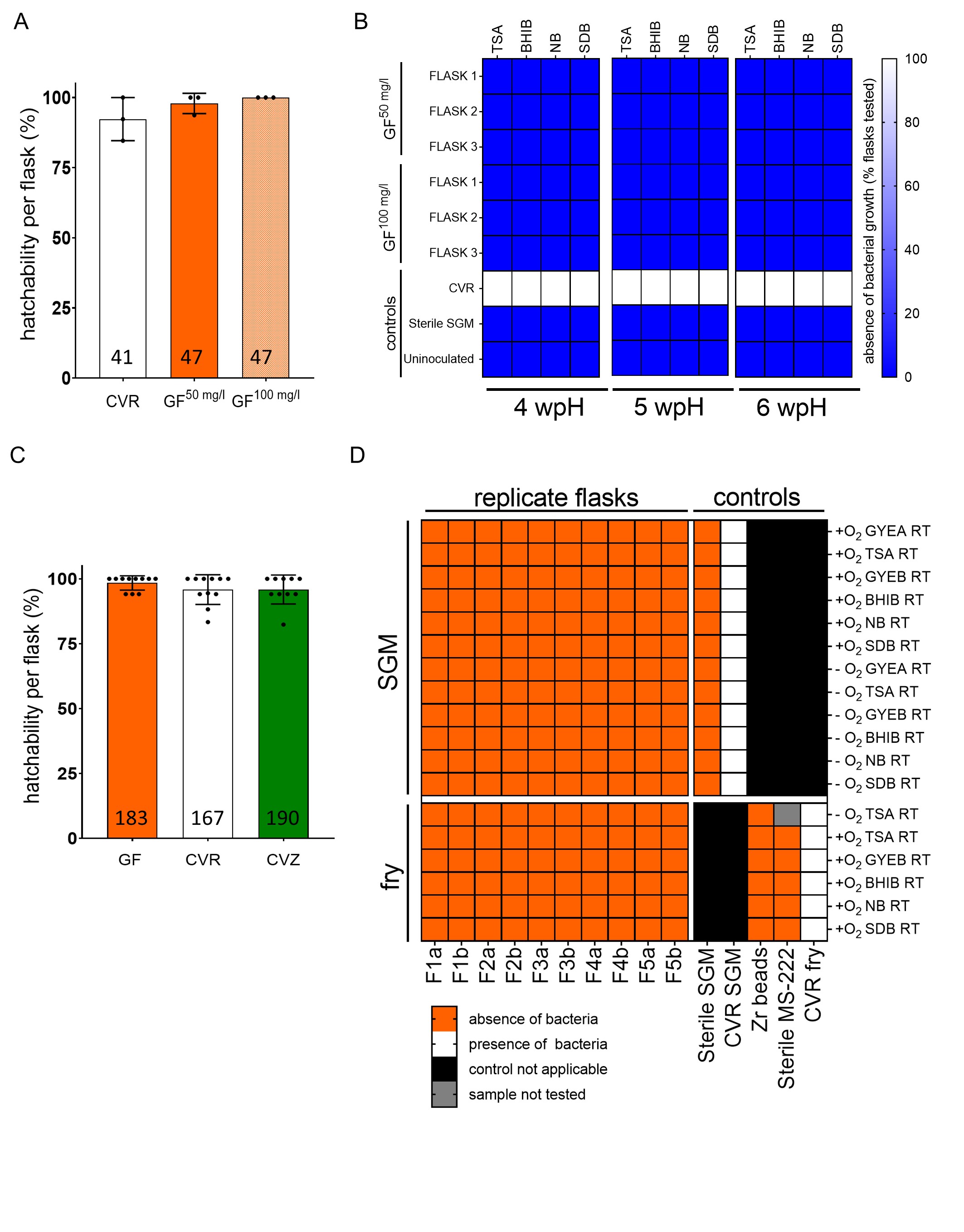

Supplement: Supplementary Figure 2 — (A) Hatchability per replicate flask at 2 wpH after two different derivation procedures (GF50mg/l and GF100mg/l). Bar graphs represent mean hatchability per treatment ± SD and the total number of eggs is reported at the bottom of each bar. Means per derivation procedure were compared to non-disinfected (conventionally raised, CVR) controls using Kruskal-Wallis and the post-hoc Dunn’s multiple comparison tests. (B) Heatmap representing the results of culture-based sterility tests, using salmon gnotobiotic media (SGM) as inoculum, sampled from flasks in (A) at 4 wpH, 5 wpH, and 6 wpH. The color scale represents the percentage of replicate flasks per treatment where microbial growth was absent. Columns represent the culture media: TSA, tryptic soy agar; BHIB, brain heart infusion broth; NB, nutrient broth; and SDB, Saboraud-Dextrose broth. Samples were incubated aerobically at room temperature. SGM from CVR flasks was used as positive control; uninoculated media and sterile SGM, as negative controls. (C) Hatchability per replicate flask at of non-disinfected (conventionally raised, CVR), GF, and recolonized germ-free yolk sac fry (conventionalized, CVZ) at 2 wpH. Bar graphs represent mean hatchability per microbial condition ± SD, and the total number of eggs per treatment is reported at the bottom of each bar. Means per condition were compared to each other using Kruskal-Wallis and the post-hoc Dunn’s multiple comparison tests. (D) Graphical summary of results from sterility tests of SGM and fry homogenates sampled from GF flasks at 12 wpH. Columns correspond to individual flasks and controls; and rows, to the culture media and conditions used. Culture media, oxygen condition (-O2, anaerobic; +O2, aerobic), temperature (RT, room temperature) and the relevant controls per sample type are listed in the figure (also see Materials and Methods). Color representation of results is indicated in the legend. [file Image_2.jpg]

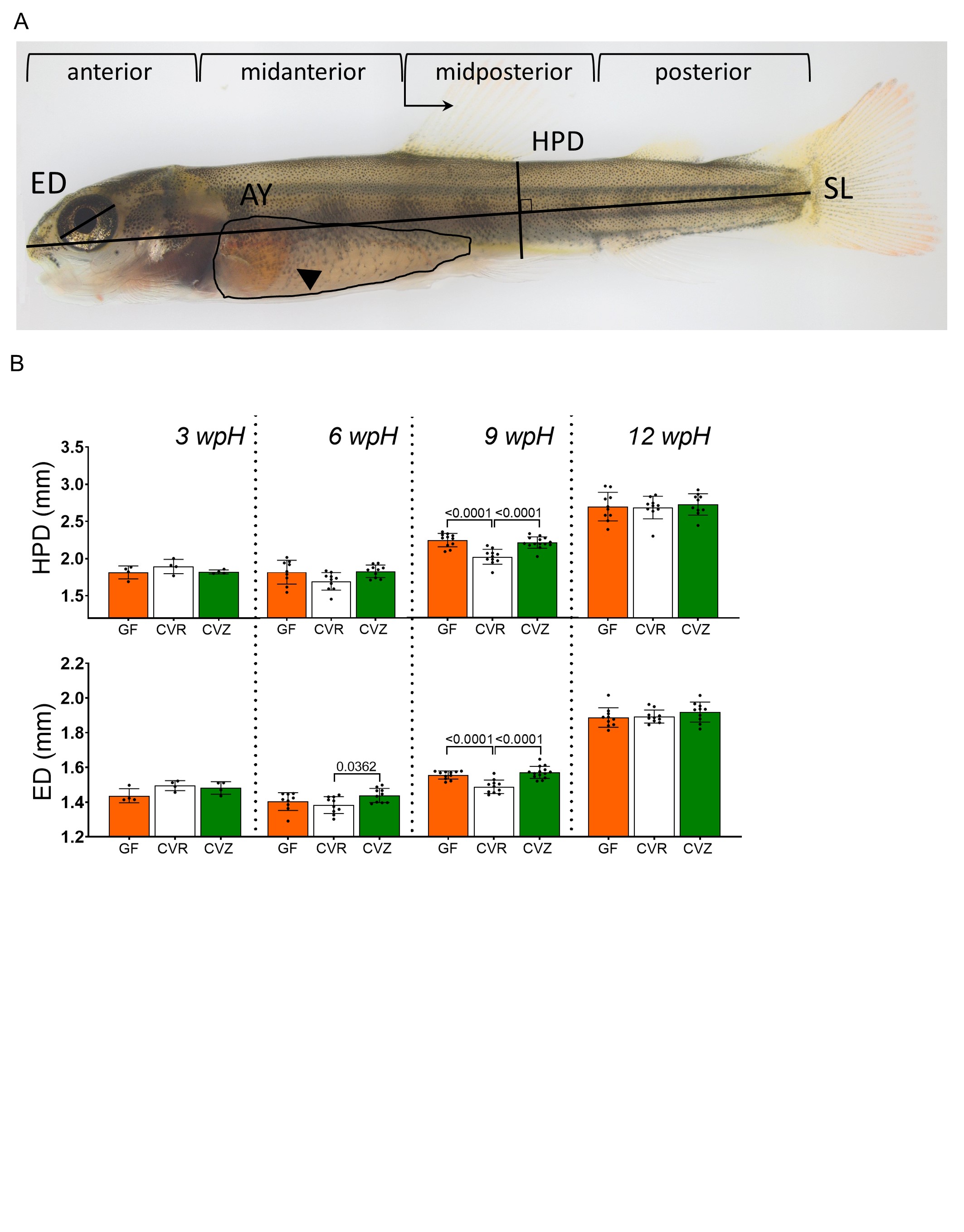

Supplement: Supplementary Figure 3 — (A) Representative micrograph of a 12 wpH CVR fry used to illustrate the morphometric characteristics measured in the analysis conducted between 3 wpH and 12 wpH. Measurements included standard length (SL), height at posterior of dorsal fin (HPD), eye diameter (ED), and area of the yolk area (AY). Arrowhead points to the single large oil globule in the yolk. Body segments sectioned for histological analysis of adipose tissue are indicated with brackets: anterior, midanterior, midposterior, and posterior. The direction of sectioning for the histology analysis in the midposterior segment is indicated. (B) Measurements of ED and HPD from fry housed flasks in GF, CVR, and CVZ flasks at 3 wpH, 6 wpH, 9 wpH, and 12 wpH. Two replicate flask were sampled per condition each timepoint. Nine to fourteen fry were sampled per condition, except at 3 wpH (see Methods). Bar graphs represent the mean of these measurements ± SD. Means were compared to each other using one-way ANOVA and post-hoc Tukey’s multiple comparison tests. [file Image_3.jpeg]
